# Contribution of quantitative viral markers to document hepatitis B virus compartmentalization in cerebrospinal fluid during hepatitis B with neuropathies

**DOI:** 10.1007/s13365-018-0662-0

**Published:** 2018-08-10

**Authors:** Charlotte Pronier, Dominique Guyader, Caroline Jézequel, Pierre Tattevin, Vincent Thibault

**Affiliations:** 1 Department of Virology, CHU Pontchaillou, Univ Rennes, INSERM, EHESP, IRSET - UMR_S 1085, Rennes, France; 2 Deparment of Liver diseases (SMF), Pontchaillou University Hospital, Univ Rennes, UMR 1241, Rennes, France; 3 Infectious Diseases and Intensive Care Unit, Pontchaillou University Hospital, Rennes, France

**Keywords:** Hepatitis B, Cerebrospinal fluid, Quantitative HBs antigen, Viral load, Extrahepatic manifestation

## Abstract

Extrahepatic manifestations linked to hepatitis B Virus (HBV) are usually indirect consequences of immune-mediated mechanisms triggered by the virus replication. Strong evidence of brain HBV replication is missing and direct involvement of HBV in nervous system symptoms has been rarely reported. We report two cases of neurological manifestations contemporary to HBV infection. In both cases, HBV-DNA and HBsAg could be quantified in the cerebrospinal fluid (CSF) at relatively high levels. Differential quantification of HBsAg and HBV viral load both in CSF and in blood as well as phylogenic studies on HBV genomic sequences obtained from blood and CSF provided strong arguments for central nervous system viral replication in both cases. Direct causality of HBV replication in the central nervous system in these clinical situations is certainly not demonstrated but these findings could expand the list of hepatitis viruses possibly involved in neurological disorders. Further studies should be promoted to better document possible HBV replication in the brain tissues and its consequences.

**Electronic supplementary material:**

The online version of this article (10.1007/s13365-018-0662-0) contains supplementary material, which is available to authorized users.

Hepatitis viruses’ (A to E) main characteristic is obviously their hepatotropism and the immune-induced liver cytolysis sometimes observed after infection. Yet, at least for HCV and HEV, diverse central nervous system manifestations have been described reflecting potential viral replication in neuronal cells (Dalton et al. 2017; Iriana et al. 2017). During HBV infection, a broad range of extrahepatic manifestations has been reported, including polyarteritis nodosa and glomerulonephritis, as well as various neurological and dermatologic diseases. The pathophysiology of HBV-associated extrahepatic manifestations is deemed to result mostly from secondary immune complex reactions; however, extrahepatic viral replication with potential direct virus effects has also been suggested (Mason et al. 1993).

We report two cases of neurological manifestations of acute onset, contemporary to HBV detection in the central nervous system.

## Case report 1

A 55-year-old male patient (A) was admitted in the emergency department for facial palsy, diplopia, and ataxia. Past medical history was unremarkable. Ten days before, he developed jaundice, arthromyalgia, light-colored stools, and dark urine. Physical examination on admission was remarkable for cutaneous and scleral icterus, facial palsy, and cerebellar syndrome. Laboratory values are presented in Table 1. MRI revealed hyper intense signal in the postero-lateral part of the right pons. Cerebrospinal fluid (CSF) basic analyses were normal. After exclusion of other viral hepatitis causes, final diagnosis was acute hepatitis B (Table 1), although no infection risk factor was identified. Neurological symptoms resolved spontaneously over 2 weeks, liver function tests normalized within 4 weeks, and serological follow-up indicated HBs seroconversion and undetectable HBV viral load (HBV-VL) by PCR in plasma. To assess the possible involvement of HBV in transient neurological disorders, HBsAg level (DiaSorin LIAISON® XL Murex HBsAg Quant) and HBV-VL (Abbott RealTime HBV-DNA) were measured in parallel in plasma and CSF. Surprisingly, both markers could be quantified in the CSF despite the absence of red blood cell, excluding significant blood contamination in CSF. The ratio of HBsAg to HBV-VL (HBsAg/HBV-VL) was 0.79 in blood, as compared to 0.0079 in CSF, which suggests different dynamics in both compartments (Table 1).

**Table 1:** Laboratory values

Patient	A	B
Sex	M	M
Age (years)	55	88
Liver function tests		
ALT (IU/L, normal values < 35)	3133	59
AST (IU/L, normal values < 45)	1788	61
Bilirubin (μM, normal values < 34)	241	11
INR	1.12	1.21
CSF analysis		
Protein (g/L)	0.42	0.69
Erythrocytes/mL	0	6
White blood cells/mL	4	3
Serology		
Anti-HBc IgM	POS	NEG
Anti-HBc IgG	NEG	POS
HBe Ag	POS	POS
Anti-HBe Ab	NEG	NEG
Anti-HDV Ab	NEG	NEG
HBV markers		
Genotype	A2	A2
Plasma HBV-VL (log_10_) IU/mL	18,983 (4.28)	250,940,372 (8.40)
Serum HBsAg (log_10_) IU/mL	15,000 (4.18)	39,000 (4.59)
CSF HBV-VL (log_10_) IU/mL	280 (2.44)	1000 (3)
CSF HBsAg (log_10_ ) IU/mL	2.22 (0.34)	7.3 (0.86)
Plasma HBsAg/HBV-VL ratio	0.79	0.00015542
CSF HBsAg/HBV-VL ratio	0.0079	0.0073
Blood/CSF VL ratio	68	250,940
Blood/CSF HBsAg ratio	6757	5342

## Case report 2

An 88-year-old male patient (B) was admitted after a recent fall at home and cognitive disorders that developed over the last 2 years with progressive loss of autonomy associated with pruritus. He was diagnosed with chronic hepatitis B (Table 1), despite no reported recent risk factor. Liver ultrasound examination was normal. Etiology of cognitive disorders remained undocumented despite comprehensive investigations, including brain MRI, and CSF analysis. As for case no. 1, direct markers of HBV replication were positive in CSF in the absence of significant contamination by blood, and HBsAg/HBV-VL ratio were 365 higher in CSF (0.0073), than in blood (0.00002). Eighteen months later, he is still alive, with no progression of baseline neurocognitive disorders.

## Discussion

These two observations are remarkable by the documentation of specific HBV marker profiles in the central nervous system compartment, in association with neurological symptoms of unknown origin. Of note, lumbar puncture was not motivated by HBV infection, but as part of the diagnostic work out of unexplained neurological disorders. Very few reports using sensitive methods have focused on HBV markers in this compartment and, to our knowledge, HBsAg has never been quantified in the CSF.

Ene et al. have recently described HBV compartmentalization in the CSF of 26 patients co-infected with HIV and HBV (Ene et al. 2015). In their study, one argument for in situ replication of HBV was the differential blood over CSF viral replication ratio between HBV and HIV. The two cases reported herein, not HIV co-infected, had strikingly different blood/CSF ratios for HBV-VL, while HBsAg ratios were comparable (Table 1). HBV morphogenesis is characterized by the release of large amount of subviral particles (surface antigen particles, genome-free virions, RNA-containing particles) besides complete virions. Thus, the HBsAg over VL (often reported as HBV-DNA) ratio indirectly reflects the amount of subviral particles over DNA containing Dane particles, as they are overproduced by a factor of 10 to 10,000 during the different phases of the disease (Désiré et al. 2015). In the two cases reported herein, with different blood viral loads and HBsAg concentrations, it is interesting to note that HBsAg over VL ratios were comparable in CSF while very different in blood. This suggests that quantification of HBV markers in CSF does not merely reflect blood-to-CSF passage, but rather a dynamic process implying viral production from different sites. HBV replication has been described in various compartments or organs. However, due to the very high replication level of this virus in the blood, it has always been difficult to rule out an artifact effect due to blood contamination. In CSF, significant blood contamination is unlikely when erythrocytes counts are < 10/mm^3^, as in our observations.

The link between HBV detection in CSF and neurological manifestations remains to be proven. Indeed, previous reports on neurologic disorders during the course of hepatitis B have raised several hypotheses such as disruption of the blood-brain barrier and/or generation of hepatitis B immune complexes to explain the presence of HBV material in CSF (Penner et al. 1982; Plough and Ayerle 1953; Stübgen 2011; Tsukada et al. 1987; Yimam et al. 2013). Unequal transfer of virions and/or subviral particles through the blood-brain barrier could also be an alternative explanation to our findings. Unfortunately, residual CSF from the described two patients was too low to perform further investigations such as immune-complexes characterization. Additional measurements to support the immunopathogenesis of HBV-related neurologic symptoms would have been true added values.

Little is known about specific cell types that would permit viral replication in the central nervous system, although neuronal cells and monocytes were found to be positive for HBV replicative intermediates (Mason et al. 1993). Yet, if HBV replication occurs within the central nervous system, unresolved issues remain, including neuronal cell HBV permissiveness and the NTCP receptor involvement, a key molecule in HBV cell infection (Watashi et al. 2014).

**Fig. 1:**
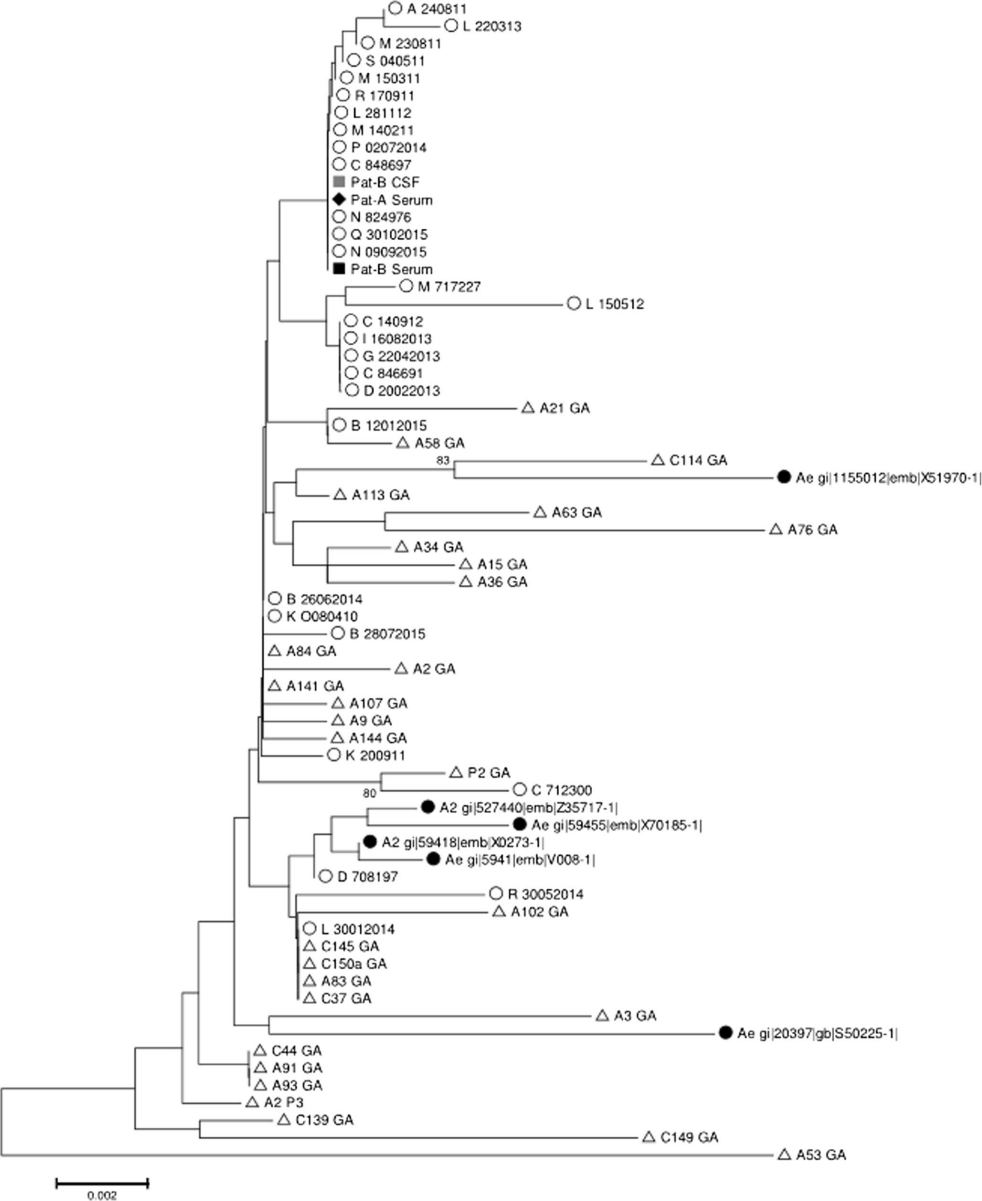
Phylogenetic tree of 721 nucleotide polymerase coding HBV genotype A sequences (*n* = 67). Open circle: not related randomly selected local sequences (*n* = 29); open triangle: acute French hepatitis B (*n* = 29); closed circle: Genbank reference sequences (*n* = 6); closed losange: patient A blood strain; closed square and gray square: patient B blood and CSF strains, respectively. The evolutionary distances were computed using the Jukes-Cantor method. The percentage of replicate trees in which the associated taxa clustered together in the bootstrap test (500 replicates) is shown next to the branches when above 70%

Phylogenic analysis indicates that all patient polymerase/HBsAg encoding sequences (nt. 271-1022) clustered with local genotype A2 strains from other patients with acute hepatitis B without neurologic symptoms (Fig. 1). Plasma and CSF strains from patient B were also identical, ruling out a genetically driven compartmentalization in CSF. Noteworthy and as described by Inoue et al., cloning analysis or next-generation sequencing may have revealed subtle genetic differences between both compartments (Inoue et al. 2008).

In conclusion, we report two cases of neurological symptoms associated with HBV replication in the central nervous system, an observation which is somewhat reminiscent of recent work on HEV (Dalton et al. 2017). Yet, any link between CSF HBV material detection and neurological disorders should certainly not be claimed from these observations but they open the possibility of an HBV replication site or a reservoir within the neural tissue that remains to be identified. More studies should now focus on a potential link between HBV replication in the central nervous system and neurological symptoms.

## Electronic supplementary material

ESM 1 (PNG 47 kb)
