# Supplementary figures and images for: Contribution of quantitative viral markers to document hepatitis B virus compartmentalization in cerebrospinal fluid during hepatitis B with neuropathies
Source: J Neurovirol. 2018 Aug 10;24(6):769–72. doi: 10.1007/s13365-018-0662-0 (PMC6280805; doi:10.1007/s13365-018-0662-0)

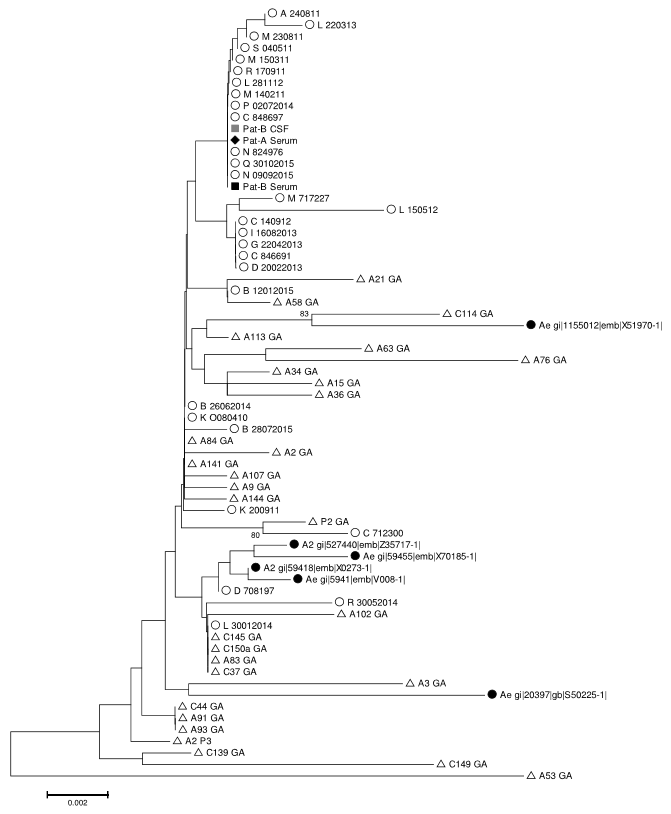

Supplement: Supplementary file 1 — (PNG 47 kb) [file 13365_2018_662_MOESM1_ESM.png]
